# Asthma prevalence among United States population insights from NHANES data analysis

**DOI:** 10.1038/s41598-024-58429-5

**Published:** 2024-04-05

**Authors:** Sarya Swed, Bisher Sawaf, Feras Al-Obeidat, Wael Hafez, Amine Rakab, Hidar Alibrahim, Mohamad Nour Nasif, Baraa Alghalyini, Abdul Rehman Zia Zaidi, Lamees Alshareef, Fadel Alqatati, Fathima Zamrath Zahir, Ashraf I. Ahmed, Mulham Alom, Anas Sultan, Abdullah AlMahmoud, Agyad Bakkour, Ivan Cherrez-Ojeda

**Affiliations:** 1 Faculty of Medicine, Aleppo University, Aleppo, Syria; 2 Department of Internal Medicine, Hamad Medical Corporation, Doha, Qatar; 3 Associate Professor at the College of Technological Innovation at Zayed University, Abu Dhabi - Khalifa City, FF2-0-032; Abu Dhabi Campus, Abu Dhabi, UAE; 4 NMC Royal Hospital, 16Th Street, Khalifa City, Abu Dhabi, UAE; 5 Assistant Professor; Internal Medicine Department, Medical Research and Clinical Studies Institute,, The National Research Centre, 33 El Buhouth St, Ad Doqi, Dokki, Cairo Governorate 12622, Cairo, Egypt; 6 Weill Cornell Medical College, Doha, Qatar; 7 Department of Family and Community Medicine, College of Medicine, Alfaisal University, Riyadh, Saudi Arabia; 8 Faculty of Medicine, Qatar University, Doha, Qatar; 9 Southern Illinois University School of Medicine, Springfield, IL USA; 10 Department of Internal Medicine, Pulmonary Division, King Faisal Specialist Hospital and Research Centre, Jeddah, Saudi Arabia; 11 Internal Medicine, Gastroenterology, Hepatology; Dr. Soliman Fakeeh Hospital (DSFH), Jeddah, Saudi Arabia; 12 Sheikh Khalifa Medical City, Abu Dhabi, UAE; 13 Universidad Espíritu Santo, Samborondón, Ecuador; 14 Respiralab Research Group, Guayaquil, Ecuador

**Keywords:** Asthma, NHANES, U. S, Asthma, Diseases

## Abstract

Asthma is a prevalent respiratory condition that poses a substantial burden on public health in the United States. Understanding its prevalence and associated risk factors is vital for informed policymaking and public health interventions. This study aims to examine asthma prevalence and identify major risk factors in the U.S. population. Our study utilized NHANES data between 1999 and 2020 to investigate asthma prevalence and associated risk factors within the U.S. population. We analyzed a dataset of 64,222 participants, excluding those under 20 years old. We performed binary regression analysis to examine the relationship of demographic and health related covariates with the prevalence of asthma. The study found that asthma affected 8.7% of the U.S. population. Gender emerged as a significant factor, with 36.0% of asthma patients being male and 64.0% female (p < 0.001). Individuals aged 60 and older having the highest asthma prevalence at 34.0%. Non-Hispanic whites had the highest prevalence at 46.4%, followed by non-hispanic blacks at 26.0%. In contrast, Mexican Americans and other hispanic individuals had lower rates, at 9.6% and 9.0%, respectively. Females were 1.76 times more likely to have asthma than males (p < 0.001). Obese individuals had a 1.74 times higher likelihood of current asthma compared to underweight individuals (p < 0.001). Notably, both Non-Hispanic Whites and Non-Hispanic Blacks showed higher odds of current asthma compared to Mexican Americans (with adjusted odds ratios of 2.084 and 2.096, respectively, p < 0.001). The research findings indicate that asthma is prevalent in 8.7% of the U.S. population. Our study highlights that individuals who are female, have low income, are obese, and smoke have the highest likelihood of being affected by asthma. Therefore, public health policies should prioritize addressing these risk factors in their preventive strategies.

## Introduction

Asthma, a chronic respiratory illness characterized by airway inflammation, constriction, and excessive mucus production^1^. Symptoms like wheezing, coughing, chest tightness, and breathlessness can severely limit daily activities, hinder physical exertion, and disrupt sleep patterns. These constraints may also impact social interactions, participation in leisure activities, and overall quality of life. Poorly managed asthma can result in decreased lung function, reduced physical activity, and declining general health^2^. Both active and passive smoking are consistently associated with an increased risk of developing and exacerbating asthma. Conversely, engaging in physical exercise has been shown to have a protective effect against asthma, while obesity and a higher body mass index (BMI) are associated with a higher susceptibility to asthma^3^. Environmental variables, including both outdoor and indoor air pollution, have also been linked to an increased risk of developing asthma and worsening symptoms. Certain occupational exposures to irritants, chemicals, and allergens may also heighten the risk of asthma development^4^. The presence of genetic predisposition, coupled with a family history of asthma or allergies, increases the likelihood of developing asthma^5^. Costs associated with asthma-related healthcare are substantial, covering expenses for prescriptions, hospitalizations, emergency room visits, and outpatient care. Asthma prevalence is influenced by demographic factors such as age, gender, race, and ethnicity^6,7^. Research has revealed noteworthy variations in asthma occurrence among different ethnic groups, with Asian Indians and Chinese individuals exhibiting lower rates (4–5%) compared to Puerto Ricans, who have the highest frequency (19%). Non-Hispanic whites and other minority groups fall within the moderate range^8^. This global health concern affects millions of individuals and presents a major challenge in the realm of public health. Notably, there has been a consistent increase in asthma prevalence within the United States, underscoring the need for a more comprehensive understanding of its occurrence among adults. A previous study, based on data from the National Health and Nutrition Examination Survey (NHANES) conducted between 2013 and 2014, reported a prevalence of approximately 8.3%^9^.

The World Health Organization (WHO) reports that the incidence of asthma in individuals between the ages of 18 and 45 varies by nation. Australia has the highest rate of any country, with 21.5%; Sweden is next with 20.2%, the UK with 18.2%, the Netherlands with 15.3%, and Brazil with 13.0%. On the other hand, China, Vietnam, and Bosnia-Herzegovina record the lowest rates, at 1.4%, 1.0%, and 1.0%, respectively^10^.

The objectives of asthma management include controlling symptoms, giving immediate relief, and reducing side effects, exacerbations, airway restriction, and asthma-related mortality. Asthma care should be personalized and dynamic, comprising constant evaluation, therapy modifications, and review, rather than relying on a strict, standardized approach^11^. The availability of an asthma action plan helps patients manage their illness proactively, allowing for prompt treatments and optimizing asthma management^12^. Acquiring knowledge on the incidence and trends of asthma in the American population is essential for establishing preventative and treatment measures, allocating resources wisely, and making well-informed choices about public health. Using the NHANES dataset, we carried out a thorough study to get an accurate estimate of the prevalence of asthma in the United States.

## Methods

### Study design

Data from the National Health and Nutrition Examination Survey (NHANES) between 1999 and 2020 was utilized for estimating the prevalence of asthma among U.S population and determine its risk factors. NHANES is a comprehensive survey conducted by the National Center for Health Statistics (NCHS) to assess the relationship between nutritional status, health promotion, and disease prevention^13^. The survey encompasses interviews and physical examinations conducted by trained medical professionals^13^. NHANES was approved by the National Center for Health Statistics research ethics review board (https://wwwn.cdc.gov/Nchs/Nhanes/). Written informed consent was obtained from all participants. All methods were performed in accordance with the relevant guidelines and regulations.

### Participant selection

The initial participant pool for this analysis included 116,876 individuals who participated in NHANES surveys. We excluded individuals under the age of 20, which reduced the sample size to 64,313 individuals. Subsequently, participants with missing data on asthma were excluded, further reducing the sample to 64,250 individuals. Additionally, participants with missing data on emphysema and chronic bronchitis were also excluded, resulting in a final sample size of 64,222 individuals available for analysis.

### Inclusion and exclusion criteria

The inclusion criteria for this study encompassed individuals who participated in NHANES surveys conducted between 1999 and 2020 and were aged 20 years or older. Exclusion criteria involved participants with missing data on asthma, and those with missing data on emphysema and chronic bronchitis.

### Interview setting and mode of administration, quality assurance & quality control

Trained interviewers conducted the inquiries at participants' residences using the computer-Assisted Personal Interview (CAPI) system. The survey questionnaire is available on the NHANES website. The CAPI system incorporates internal checks to reduce data entry errors and offers online help screens to aid interviewers in grasping essential terms in the questionnaire. After collecting the data, NHANES field office staff meticulously examined the interviews to guarantee the precision and thoroughness of specific elements. Additionally, interviewers had to document the interviews, and NCHS staff and interviewer supervisors reviewed these recorded sessions.

### Assessment outcomes

The interviewed participants were asked “Ever been told you have asthma?”, and the responses were “Yes”,”No”,”Don’t Know” and “Refused”.

### Covariates

In our survey, we collected and analyzed various covariates. These included demographic factors such as gender (categorized as Male or Female), age (grouped into 20–29, 30–39, 40–49, 50–59, and 60 +), and race/ethnicity (classified as Mexican American, Other Hispanic, Non-Hispanic White, Non-Hispanic Black, and Another race/ethnic group). Educational background was categorized into Less Than 9th Grade, 9-11th Grade, High School Grad/GED or Equivalent, Some College or AA degree, College Graduate or above, with a category for missing values. Marital status was represented by Married, Widowed, Divorced, Separated, never married, and living with partner, along with a category for missing values. Socioeconomic status was captured through Family PIR (income), which was categorized as Low income, Middle income, High income, with a category for missing values. Health-related variables included BMI (Body Mass Index) categories: Underweight, Normal weight, Overweight, Obese, and a category for Missing value. Participants' respiratory health was assessed using variables like Shortness of Breath on Stairs/Inclines (No, yes, and Missing values), Shortness of Breath on a Level Surface (No, Yes, and Missing values), and ever being told they had conditions such as Emphysema (No, Yes) and Chronic Bronchitis (No, Yes, with a category for Missing values). Additionally, participants' smoking status was documented as Non-smoker, Past-smoker, Current-smoker, and included a category for Missing values.

### Statistical analysis

In this study, we employed a range of statistical techniques to analyze the NHANES III data. Using SPSS-26 software and incorporating sampling weights to calculate variances, our analysis commenced with descriptive statistics to characterize the study population. We utilized the Chi-square test and Kruskal–Wallis H-test to explore differences among various demographic groups. To identify the most susceptible groups, pairwise-comparison analyses were conducted.

For the investigation of influential factors that may explain asthma, we employed logistic regression models, including binary logistic regression for dichotomous dependent variables. In the final models, we included variables that were significant at p < 0.05 from the univariate analysis. Our analysis reported both crude and adjusted odds ratios (OR) along with 95% confidence intervals, providing a comprehensive understanding of the associations between the presence of asthma and other covariates. We considered statistical significance at a p-value less than 0.05.

## Results

### Sociodemographic characteristics of the included U. S individuals

The overall asthma prevalence percentage among the U.S. population was 8.7%. Among those with asthma, 36.0% were males, and 64.0% were females (p < 0.001). The age group with the highest asthma prevalence was individuals aged 60 years and older, at 34.0%, while participants aged 20–29 showed the lowest percentage (p < 0.001). Non-Hispanic Whites had the highest asthma prevalence at 46.4%, followed by non-Hispanic Blacks at 26.0% (p < 0.001). Furthermore, an observed association of significance (p < 0.001) between body mass index (BMI) and the prevalence of asthma was found to be such that obese individuals exhibited the highest prevalence of asthma at 49.5%. In contrast, underweight individuals displayed the lowest prevalence (1.5%). Smoking and asthma were significantly associated (p < 0.001), with a higher rate of asthma in smokers (51.2%) compared to non-smokers (48.4%) Among participants who reported having emphysema, a notably higher proportion (9.1%) also had asthma, in contrast to those without asthma (1.4%) (p < 0.001). A similar trend is evident in participants reporting chronic bronchitis, where individuals with asthma exhibited a significantly higher prevalence (16.4%) compared to those without asthma (1.6%). Moreover, participants with asthma demonstrated a higher prevalence of COPD, emphysema, or chronic bronchitis (COPD, Emphysema, ChB) (31.1%) than their counterparts without asthma (6.8%), with a highly significant p-value (p < 0.001) (Table 1).

**Table 1:** Sociodemographic characteristics of the included U. S individuals.

Variables	Total	With asthma	Without asthma	P value
Subjects	64,222	5566 (8.7)	58,656 (91.3)	
Gender				0.000
Male	30,901 (49.1)	2006 (36.0)	28,895 (49.3)	
Female	33,321 (51.9)	3560 (64.0)	29,761 (50.7)	
Age (years)				0.000
20–29	10,927 (17.0)	1026 (18.4)	9901 (16.9)	
30–39	10,677 (16.6)	839 (15.1)	9838 (16.8)	
40–49	10,502 (16.4)	867 (15.6)	9635 (16.4)	
50–59	9654 (15.0)	930 (16.7)	8724 (14.9)	
60 +	22,462 (35.0)	1904 (34.)	20,558 (35.0)	
Race/ethnicity				0.000
Mexican–American	10,634 (16.6)	533 (9.6)	10,101 (17.2)	
Other Hispanic	5455 (8.5)	499 (9.0)	4955 (8.4)	
Non-Hispanic White	27,486 (42.8)	2585 (46.4)	24,901 (42.5)	
Non-Hispanic Black	13,955 (21.7)	1447 (26.0)	12,508 (21.3)	
Other race/ethnic group	6692 (10.4)	502 (9.0)	6190 (10.6)	
Education				0.000
Less than 9th grade	7536 (11.7)	525 (9.5)	7011 (12.0)	
9–11th grade (includes 12th grade with no diploma)	9331 (14.5)	895 (16.1)	8436 (14.4)	
High school grad/GED or equivalent	14,938 (23.3)	1314 (23.7)	13,624 (23.3)	
Some college or AA degree	18,324 (28.5)	1775 (32.0)	16,549 (28.3)	
College graduate or above	13,964 (21.7)	1042 (18.8)	12,922 (22.1)	
Missing values	129 (0.2)			
Marital status				0.000
Married	33,680 (52.4)	2494 (45.4)	31,186 (53.7)	
Widowed	7171 (11.2)	674 (12.3)	6497 (11.2)	
Divorced	7375 (11.5)	842 (15.3)	6533 (11.2)	
Separated	1812 (2.8)	209 (3.8)	1603 (2.8)	
Never married	9575 (14.9)	918 (16.7)	8657 (14.9)	
Living with partner	4011 (6.2)	362 (6.6)	3649 (6.3)	
Missing values	598 (0.9)			
Family PIR				0.000
Low income (PIR < 1.3)	17,605 (17.4)	1927 (38.5)	15,678 (29.9)	
Middle income (1.3 ≤ PIR < 3.5)	22,058 (34.3)	1777 (35.5)	20,281 (38.6)	
High income (PIR ≥ 3.5)	17,861 (27.8)	1307 (26.1)	16,554 (31.5)	
Missing values	6698 (10.4)			
BMI				0.000
Underweight	957 (1.5)	79 (1.5)	878 (1.6)	
Normal weight	16,450 (25.6)	1134 (21.7)	15,316 (28.1)	
Overweight	19,987 (31.1)	1422 (27.2)	18,565 (34.1)	
Obese	22,238 (34.6)	2585 (49.5)	19,653 (36.1)	
Missing value	4590 (7.1)			
Shortness of breaths on stairs/inclines				0.000
No	27,536 (42.9)	1209 (32.8)	26,327 (67.8)	
Yes	14,961 (23.3)	2473 (67.2)	12,488 (32.2)	
Missing values	21,725 (33.8)			
Short of breath walking on level surface				0.000
No	4963 (7.7)	670 (59.1)	4293 (75.9)	
Yes	1823 (2.8)	463 (40.9)	1360 (24.1)	
Missing values	57,436 (89.4)			
Ever told you had emphysema				0.000
No	53,822 (83.8)	4230 (90.9)	49,592 (98.6)	
Yes	1131 (1.8)	423 (9.1)	708 (1.4)	
Ever told you had chronic bronchitis				0.000
No	53,355 (83.1)	3889 (83.9)	49,466 (98.4)	
Yes	1564 (2.4)	761 (16.4)	803 (1.6)	
Missing values	9303 (14.5)			
Ever told you had COPD, emphysema, ChB				0.000
No	8361 (13.0)	616 (68.9)	7745 (93.2)	
Yes	844 (1.3)	278 (31.1)	566 (6.8)	
Missing values	55,017 (85.7)			
Smoking				0.000
Non-smoker	35,327 (55.0)	2712 (48.8)	32,615 (55.7)	
Past-smoker	15,432 (24.0)	1438 (25.9)	13,994 (23.9)	
Current-smoker	13,398 (20.9)	1410 (25.4)	11,988 (20.5)	
Missing values	65 (0.1)			

### Asthma prevalence by gender and age group

Females have a higher asthma prevalence of 10.7%, while males have a lower prevalence of 6.5%. In addition, those aged 50–59 have the highest current asthma prevalence at 9.6%, while those aged 30–39 have the lowest prevalence at 7.9% (Table 2).

**Table 2:** Asthma prevalence by gender and age group.

	Asthma	
	Unweighted n	Weighted %
Gender		
Male	2006	6.5
Female	3560	10.7
Age (years)		
20–29	1026	9.4
30–39	839	7.9
50–59	930	9.6
60 = <	1904	8.5

### Logistic regression analysis of asthma risk factors

We used two models—an adjusted model and an unadjusted model—that included gender, age groups, race, educational attainment, marital status, PIR categories, BMI groups, and smoking to determine the predictive values of having asthma in relation to all co-variables. Asthma was more common in women than in men (AOR = 1.76, p < 0.001). Additionally, compared to underweight people, obese participants had a higher likelihood of having asthma (AOR = 1.74, p < 0.001). Individuals aged 60 and older were less likely to develop asthma than those aged 20 to 29 (COR: 0.894, AOR: 0.869, P-value 0.05). Individuals with higher incomes were less likely to have asthma than those with lower incomes (AOR = 0.683, p < 0.001). The non-Hispanic White and non-Hispanic Black participants had greater chances of asthma than the Mexican American participants (AOR = 2.084 and 2.096, respectively, p < 0.001). Furthermore, current smokers had a higher odd of asthma than non-smokers (AOR = 1.36, COR = 1.414, p < 0.001) (Table 3).

**Table 3:** Estimated crude and adjusted odds ratios (OR) and 95% confidence intervals (CI) using current asthma and wheezing in the previous12 months as an outcome in the National Health and Nutrition Examination Survey.

Variables	Crude, OR, [95% CI]	Adjusted, OR, [95% CI]
Gender		
Male	1.00	1.00
Female	1.73, [1.627–1.824]***	1.76, [1.64–1.87]***
Age (years)		
20–29	1.00	1.00
30–39	0.823, [0.748–0.905]***	0.831, [0.74–0.92]*
40–49	0.868, [0.79–0.955]*	0.847, [0.75–0.94]*
50–59	1.029, [0.937–1.129]	0.963, [0.86–1]
60 +	0.894, [0.825–0.968]**	0.869, [0.78–0.96]*
Race/ethnicity		
Mexican–American	1.00	
Other Hispanic	1.908, [1.681, 2.166]***	1.874, [1.624–2.164]***
Non-Hispanic White	1.967, [1.787, 2.166]***	2.084, [1.859–2.336]***
Non-Hispanic Black	2.192, [1.978, 2.430]***	2.096, [1.859–2.364]***
Other race/ethnic group	1.537, [1.355, 1.743]***	1.851, [1.599–2.143]***
Education		
Less than 9th grade	1.00	1.00
9–11th grade (includes 12th grade with no diploma)	1.417, [1.266, 1.585]***	1.085, [0.950–1.240]
High school grad/GED or equivalent	1.288, [1.159, 1.431]***	0.982, [0.864–1.116]
Some college or AA degree	1.432, [1.294, 1.585]***	1.122, [0.989–1.273]
College graduate or above	1.077, [0.966, 1.201]	1.037, [0.901–1.193]
Marital status		
Married	1.00	1.00
Widowed	1.297, [1.187, 1.418]***	1.032, [0.925–1.151]
Divorced	1.162, [1.484, 1.75]***	1.285, [1.171–1.410]*
Separated	1.63, [1.404, 1.894]***	1.277, [1.078–1.512]*
Never married	1.326, [1.225, 1.435]***	1.136, [1.030–1.252]*
Living with partner	1.241, [1.105, 1.392]***	1.101, [0.967–1.255]
Family PIR		
Low income (PIR < 1.3)	1.00	1.00
Middle income (1.3 ≤ PIR < 3.5)	0.713, [0.666, 0.763]***	0.724, [0.673–0.780]***
High income (PIR ≥ 3.5)	0.642, [0.597, 0.691]***	0.683, [0.625–0.747]***
BMI		
Underweight	1.00	1.00
Normal weight	0.823, [0.649, 1.044]	0.96, [0.75–1.24]
Overweight	0.851, [0.672, 1.078]	1.10, [0.86–1.42]
Obese	1.462, [1.157, 1.847]**	1.74, [1.36–2.24]***
Shortness of breaths on stairs/inclines		–
No	–	–
Yes	–	–
Short of breath walking on level surface		–
No	–	–
Yes	2.18, [1.91, 2.49]***	–
Ever told you had emphysema		–
No	1.00	–
Yes	7.01, [6.19, 7.93]***	–
Ever told you had chronic bronchitis		–
No	1.00	–
Yes	12.05, [10.86, 13.38]***	–
Smoking		
Non-smoker	1.00	1.00
Past-smoker	1.236, [1.156, 1.321]***	1.350, [1.24–1.45]***
Current-smoker	1.414, [1.322, 1.514]***	1.36, [1.25–1.47]***

*AOR* adjusted odds ratio, *COR* curve odds ratio.

*p < 0.05.

**p < 0.01.

***p < 0.001.

## Discussion

### Overall asthma prevalence

Asthma is a prevalent long-term respiratory disease that impacts millions of people across all age groups in the US, with an approximate 8–9% prevalence rate^14^. According to our estimated investigation, 8.7% of the population in the United States suffers from asthma. This percentage has varied in prior U. S publications, ranging from less than 3% to more than 20%^15–18^. Asthma prevalence varies by nation and is impacted by a variety of variables, including genetics, environmental circumstances, healthcare infrastructure, and lifestyle.

More than 8 million individuals in the UK, or almost 12% of the total population, have an asthma diagnosis. Nevertheless, while some individuals may have outgrown the condition, 5.4 million are currently undergoing treatment for asthma^19^. In Australia, the prevalence of asthma is 11%, much higher than the worldwide incidence of 4%^20^. In 2011–12, 3.8 million Canadians (or 10.8% of the population) had been diagnosed with asthma^21^. In Sweden, 8.3% of people had a physician-diagnosed case of asthma^22^. Most asthma cases in the Gulf States are reported from Saudi Arabia^23^. Asthma affects around 24% of the population^23^. The rates for Kuwait and Qatar are 16.8% and 19.8%, respectively^23^. This is followed by 13% in the UAE and 10% in Oman^23^. In adults in Ireland^24^, the frequency is 7%, whereas in children, it is 21%. In 2018, there were 2.4% of people in Indonesia^25^ who had asthma, and 57.5% of those people had relapses. In Africa^26^, the average asthma prevalence was 6%.

Then, our investigation prevalence approximates developed countries' reports and discovered that the reported asthma prevalence is higher in developed countries than in developing countries, explaining that in developed countries, there may be more asthma awareness, better access to healthcare, and more robust healthcare systems. Conversely, asthma may go undiagnosed and untreated in underdeveloped nations for a variety of reasons, such as lack of knowledge, restricted healthcare access, and financial limitations.

### Asthma prevalence by gender

The results of our research showed that the prevalence of asthma has significantly increased among females. Almost studies and reports found the same result^27–29^, which could be explained by the effects of female sex hormones on lung cells, and changes in hormone levels during puberty, menstruation, pregnancy, and menopause can affect airway function, which among women with asthma, up to 30–40% have reported worsening of asthma symptoms at specific times of the menstrual cycle in some series^30^. Furthermore, research indicates that women often have narrower airways, which may increase their vulnerability to airflow blockage and asthma symptoms^31^. Additionally, since women spend more time indoors, they may be more susceptible to interior allergens, especially if they have domestic duties. Dust mites, pet dander, and mold are examples of indoor allergens that may aggravate asthma symptoms.

### Asthma prevalence by age

Our findings of increased asthma prevalence among the age groups 50–59 and 20–29 may reflect the presence of both early-onset and late-onset asthma within these populations [AOR = 0.96, 1, respectively]. Younger adults (20–29) may predominantly exhibit early-onset asthma, characterized by allergic sensitization and environmental exposures commonly associated with young adulthood. Meanwhile, middle-aged adults (50–59) may represent a population with a higher prevalence of late-onset asthma, which tends to be more severe and non-allergic in nature.

It is widely acknowledged that early-onset asthma, typically observed in childhood or early adulthood, is often associated with atopic sensitization and allergic triggers^32,33^. This phenotype is commonly characterized by a history of allergic rhinitis or eczema, elevated levels of immunoglobulin E (IgE), and responsiveness to allergen-specific therapies^34^. In contrast, late-onset asthma tends to be more heterogeneous, with a higher prevalence of non-allergic triggers and a greater likelihood of severe symptoms^35^. Furthermore, late-onset asthma has been linked to a decline in lung function and is frequently associated with comorbidities such as obesity, chronic rhinosinusitis, and gastroesophageal reflux disease^36,37^.

### Asthma prevalence by race

Non-Hispanic whites and Non-Hispanic Black exhibited the highest asthma prevalence, while the lowest asthma prevalence indicated among Mexican Americans [AOR = 2.084, 2.096, respectively]. These results and align with our findings some studies in California, New Mexico, and Arizona had reported a lower risk of asthma and respiratory diseases among Mexican–American adults^38,39^. Genetic and biological differences among ethnic groups can influence their susceptibility to asthma. Certain genetic variations may make individuals prone to developing asthma or experiencing asthma-related symptoms^40^. Cultural practices, dietary habits, and health behaviors can vary among ethnic groups and impact asthma risk^41^. For example, dietary choices that include an abundance of fruits and vegetables, common among some Hispanic populations, may have protective effects against asthma. In addition, the migration patterns of different ethnic groups can play a major role in the difference of asthma prevalence^42^.

### Asthma prevalence by marital status

Our analysis revealed that divorced, separated and never married individuals reported a higher chance of getting asthma compared to married individuals [AOR = 1.285, 1.277, 1.136, respectively]. Contrary to our results, some studies have not found a significant difference in asthma prevalence between married individuals and their unmarried counterparts^43,44^. The interplay between marital status and asthma is multifaceted, encompassing various lifestyle and environmental factors that warrant further exploration. Marriage often provides emotional support and a sense of security, which can buffer against stressors known to exacerbate asthma symptoms. Divorced, separated, and never married individuals may experience higher levels of psychosocial stress due to lack of spousal support or social networks, which could contribute to the development or worsening of asthma. Divorced, separated, and never married individuals may experience higher levels of psychological distress, depression, or anxiety compared to married individuals. These psychological factors have been associated with increased asthma risk and exacerbation. Stress-related mechanisms, such as dysregulation of the immune system and increased inflammation, may contribute to the observed higher likelihood of asthma in these populations.

### Asthma prevalence by family income

Our analysis reported high asthma prevalence among those with low income. Studies consistently demonstrate that asthma is more prevalent among individuals with lower household incomes^45,46^. Factors like substandard housing, exposure to environmental pollutants, and limited access to healthcare can contribute to this disparity. Lower-income individuals often face disparities in healthcare access and quality, which can lead to inadequate management of asthma and increased prevalence^47^. They may have limited access to preventative care, medications, and asthma education. Low-income communities are more likely to be exposed to environmental triggers for asthma, such as air pollution, allergens, and tobacco smoke^48^. These exposures can exacerbate asthma symptoms and contribute to higher prevalence rates.

### Asthma prevalence by BMI

On this study, the highest asthma prevalence was observed among obese individuals. Numerous studies have observed a positive association between obesity, particularly higher BMI, and asthma prevalence^49,50^. Obesity is characterized by chronic low-grade inflammation, with the adipose (fat) tissue serving as an active source of pro-inflammatory molecules. This inflammatory state can extend to the airways, leading to airway inflammation. This systemic inflammation can exacerbate the inflammatory processes associated with asthma, making it more likely for asthma to develop and become more severe in obese individuals^51^. Increased fat in the chest and abdomen can limit the expansion of the lungs and diaphragm. This decreased lung volume can lead to reduced airflow and increased airway resistance, which are characteristic features of asthma^52^.

### Asthma prevalence by smoking

Higher prevalence of asthma was observed among smokers in this study. Previous studies have consistently shown that both active smoking (smoking cigarettes) and exposure to secondhand smoke (passive smoking) are associated with an increased risk of developing asthma^53,54^. Cigarette smoke contains numerous harmful chemicals and irritants that can directly damage the airways^55^. These irritants can trigger inflammation in the respiratory system, leading to airway constriction and mucus production. Smoking generates oxidative stress in the lungs, which can damage cells and tissues^56^. This oxidative stress can contribute to airway inflammation and bronchial hyperreactivity, key characteristics of asthma^56^. Smoking can disrupt the immune system's balance, leading to an enhanced response to allergens and respiratory infections. Exposure to secondhand smoke can make individuals, especially children, more sensitive to environmental allergens and respiratory irritants^57^. This heightened sensitivity can increase the likelihood of asthma development or exacerbation.

### Asthma prevalence by other respiratory conditions

Most of the asthma patients in this study suffered from other respiratory conditions like emphysema, chronic bronchitis, and COPD. According to previous study, asthma frequently coexists with other respiratory diseases^58,59^. This coexistence can complicate the management of these conditions and lead to poorer health outcomes^58^. Asthma, emphysema, chronic bronchitis, and COPD share common risk factors such as smoking, exposure to environmental pollutants, and genetic predisposition^60^. Smoking is a well-established risk factor for emphysema, chronic bronchitis, and COPD^61^. It is also associated with an increased risk of developing and exacerbating asthma^61^. The structural changes in the airways, such as airway remodeling, are common features of asthma and COPD^62^. These changes can result in increased airway hyperresponsiveness and decreased lung function, contributing to symptoms in both conditions. Emphysema also involves structural damage to the lungs.

### Clinical implications

The variation in asthma prevalence across different countries highlights the need for global awareness and standardized healthcare practices. Developed nations with higher asthma prevalence may serve as models for effective asthma management, emphasizing the importance of awareness, access to healthcare, and robust healthcare systems in reducing the burden of asthma.

Understanding the impact of hormonal changes on airway function is crucial. Tailoring treatment plans and education programs to address the unique challenges faced by women, such as hormonal fluctuations and increased exposure to indoor allergens, can improve asthma outcomes.

Recognizing the impact of immunosenescence and inflammaging on asthma symptoms is crucial. Regular monitoring, personalized treatment plans, and awareness campaigns targeting the elderly can enhance asthma care.

The strong association between obesity and asthma prevalence underscores the importance of incorporating weight management strategies into asthma care. Healthcare providers should prioritize weight reduction interventions, and obesity-related inflammation.

The higher prevalence of asthma among smokers highlights the critical role of smoking cessation programs in asthma prevention and management. Physicians should prioritize smoking cessation interventions, especially among vulnerable populations like children and adolescents, to reduce the risk of asthma development and exacerbation.

### Limitations

The reliance on NHANES data may raise concerns about the representativeness of the sample, potentially leading to the underrepresentation of specific subgroups within the U. S population. The cross-sectional design of the study, while informative about associations, cannot establish causality, leaving questions about the direction of relationships. Moreover, the study utilizes self-reported data, including self-reported physician diagnoses of asthma, introducing the possibility of recall bias and misclassification of asthma cases. While the analysis highlights the significance of age, shortness of breath, and respiratory conditions, it may overlook other unmeasured confounding variables. Comparative analyses with other studies, such as the European Community Respiratory Health Survey (ECRHS), offer valuable context but also pose challenges due to differences in study design and populations.

Additionally, its generalizability to other countries may be limited, given variations in culture, healthcare systems, and environmental factors. Lastly, the measurement of shortness of breath, while identified as significant, needs more detailed examination, potentially impacting the reliability of the findings. In addition, the COVID-19 pandemic forced the halting of NHANES 2019–2020 field activities in March 2020, after data had been gathered in 18 of the 30 survey sites in the 2019–2020 sample. Data from the previous cycle (2017–2018) were integrated with the obtained data, which were not nationally representative, to generate a pre-pandemic data file covering the period from 2017–March 2020. The pre-pandemic data set for March 2020 to March 2017 underwent a unique weighting procedure. Neither the 2017–2018 data alone nor the 2019–March 2020 data alone will provide nationally representative findings using these sample weights, nor are they suitable for independent studies of the 2019–2020 data. We acknowledge a limitation related to the chosen age cutoff. We opted to exclude individuals under the age of 20 from our analysis, which may have implications for the generalizability of our findings to the entire adult population.

## Conclusion

The investigation results determine that 8.7% of the population in the United States has asthma. Health policy and decision-makers, clinicians, and researchers can use the asthma statistics from the Nhanes dataset that is presented here to help design programs and action plans that address risk factors like being female, having low income, being obese, and smoking. These plans can also be used to improve the quality of care for asthma patients and lessen the burden that the condition puts on them by providing appropriate, easily accessible, and effective treatment.

## Data Availability

The datasets generated and/or analysed during the current study are available in the National Health and Nutrition Examination Survey repository, [https://wwwn.cdc.gov/nchs/nhanes/continuousnhanes/default.aspx]. All data generated or analysed during this study are included in this published article.
